# Allergic Rhinitis and Its Associated Co‐Morbidities Among Patients Attending the ENT Department at Kilimanjaro Christian Medical Center in Northern Tanzania: Cross‐Sectional Study

**DOI:** 10.1002/iid3.70130

**Published:** 2025-01-24

**Authors:** Kenneth Mlay, Gasper Temba, Adrian Matasha, Pendael Mzonge, Denis Katundu, Desderius Chussi

**Affiliations:** ^1^ Department of Medicine Kilimanjaro Christian Medical University College Moshi Tanzania; ^2^ Department of Otorhinolaryngology Kilimanjaro Christian Medical Centre Moshi Tanzania

**Keywords:** allergic rhinitis, ARIA, otorhinolaryngology, SFAR

## Abstract

**Introduction:**

Allergic rhinitis is the specific inflammation against allergen by immune defense cells on the nasal mucosa, which can lead to chronic nasal symptoms such as sneezing, itching, runny nose, and nasal congestion. It is associated with high morbidity including sinusitis, asthma, otitis media, hypertrophied inferior turbinate, and nasal polyps. Despite its complications, it remains poorly recognized and tracked.

**Methods:**

A cross‐sectional hospital‐based study was done, a total of 221 patients received Ear, Nose And Throat services at Kilimanjaro Christian Medical Center during the study period all patients with a clinical diagnosis of allergic rhinitis were captured; Data was collected using a pre‐tested coded questionnaire (Score For Allergic Rhinitis). The data was then analyzed using SPSS version 22.

**Results:**

A total of 221 patients with a clinical diagnosis of allergic rhinitis were approached in a 6 months study period, 111 (50.2%) were females, and 140 (63.4%) were residing in urban areas. The prevalence of allergic rhinitis was 23.9%. Factors such as age OR 0.12, 95% CI (0.03; 0.40), education OR 0.13, 95% CI (0.04; 0.44), occupation OR 3.75, 95% CI (1.36; 10.32), adenotonsillar hypertrophy OR 4.66, 95% CI (2.21; 9.80), and OME OR 4.11, 95% CI (1.32; 12.83) (*p* = 0.009) were found to be significantly associated with allergic rhinitis. 60.4%, Inferior turbinate hypertrophy is the leading co‐morbidity of allergic rhinitis which accounts for 64.7%.

**Conclusion:**

Allergic rhinitis is among the common health problems affecting Tanzanians. It is a commonly seen disorder in younger age ( < 15 years) which is in correlation with other studies done in Africa and worldwide.

## Graphical abstract

Allergic rhinitis, associated comorbidities

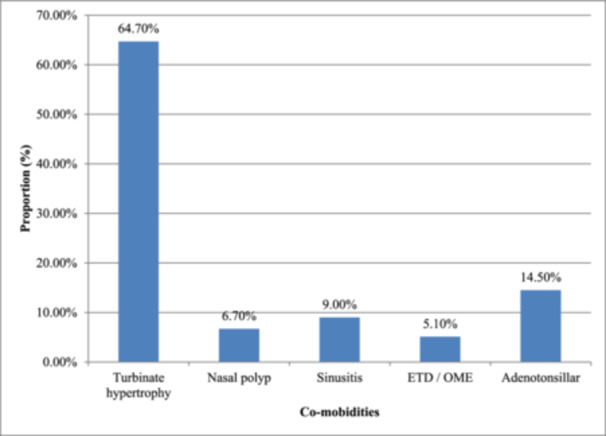

## 1 Introduction

Allergic rhinitis is the specific inflammation against allergen by immune defense cells on nasal mucosa, which can lead to chronic nasal symptoms such as sneezing, itching, runny nose, and nasal congestion [1]. Allergic Rhinitis and Its Impact on Asthma (ARIA) has a classification system that includes the categories of intermittent and persistent allergic rhinitis [2].

Allergic rhinitis is one of the most common Ear Nose and Throat conditions which has considerable effects on quality of life and can have significant consequences if left untreated [3].

The pathophysiology of AR comprises an early‐ and late‐phase allergic response. The process is triggered by exposure to allergens such as pollen, mites, and/or animal dander that are recognized by antigen‐specific immunoglobulin E (IgE) receptors on mast cells and basophils in pre‐sensitized individuals [4].

In AR, allergen‐stimulated PBMCs produce IL‐31 and correlate to the severity of the disease and its symptomatic manifestations, thus suggesting that IL‐31 may increase inflammation in the nasal epithelium through the release of mediators (CCL17, CCL22, and CCL1), which recruit inflammatory cells, as seen in Atopic dermatitis [5].

Higher levels of IL‐31 and IL‐31RA in mucosal samples of AR patients correlated with the gene expression of MUC5AC (considered to be the major mucin in the airway), providing evidence that IL‐31 also plays a role in mucus hypersecretion in AR patients.

This evidence suggests the role of IL‐31 in enhancing Th2 response and eosinophils activation in Allergic rhinitis [6].

Allergic rhinitis is characterized by a considerable medical and social burden, mainly because of its high prevalence, globally it constitutes a worldwide public health problem with a prevalence of 10% to 40% and the trend is increasing [7].

It has a major ramification in terms of associated morbidity, hence poorly controlled symptoms of allergic rhinitis may contribute to sleep loss, secondary daytime fatigue, learning impairment, decreased overall cognitive functioning, decreased long‐term productivity and reduced quality of life. It may also contribute to the development of other related disease processes including acute and chronic sinusitis, recurrence of nasal polyps, otitis media/otitis media with effusion, hearing impairment, abnormal craniofacial development, sleep apnea and related complications, aggravation of underlying asthma, and increased propensity to develop asthma [8].

If left untreated is associated with high morbidity, hence for this reason, numerous treatments have evolved over the years. Pharmacological therapy with antihistamines, decongestants, sodium cromoglycate, and corticosteroids, both topically and systemically improves symptoms in addition immunotherapy is useful in the treatment of allergic rhinitis, specifically for those patients allergic to the pollen of ragweed, grass, and trees. Most patients with seasonal allergic rhinitis are treated with either pharmacological agents or immunotherapy or a combination of the two [9].

Implementing cost‐effective therapeutic strategies is an important intervention to reduce the cost of allergic rhinitis. Although there are many economic evaluations of allergic rhinitis treatments in the published medical literature, very few represent formal cost‐effectiveness evaluations that compare alternative treatment strategies' incremental costs and benefits. No therapy that treats this serious condition can be thought of as being intrinsically cheap or expensive; it must be evaluated against the reduction in the total cost of the disease. Future work would benefit from the development of a consensus on a summary measure of effectiveness that could be used in cost‐effectiveness analyzes of therapies for allergic rhinitis [10].

One study in South Africa was done to assess the economic burden of allergic rhinitis, which reveals several burdens including direct costs such as doctor costs, medication, diagnostic tests (eg. allergy tests), Over‐the‐counter medicines, alternative complementary medicine, costs of training doctors and transport also it includes indirect costs such as loss of work days, School days lost and lastly intangible costs such as loss of quality of life, Social costs, psychological maladjustment, pain and suffering [11].

Despite the aforementioned magnitudes and co‐morbidities, allergic rhinitis remains poorly recognized and tracked due to a lack of appropriate diagnostic tools, even in settings where the test is available, only a few patients can afford the cost of the test. Availability of cost‐effective diagnostic test which can capture clients at all levels is needed, hence we conducted a study to determine the magnitude and co‐morbidities of allergic rhinitis by using a standardized questionnaire that is affordable at any level and has satisfactory sensitivity and specificity, in the absence of any allergic test [12].

## 2 Methods

### 2.1 Study Design

Hospital based descriptive cross‐sectional study which was done from January 2019 to June 2019.

### 2.2 Study Setting

The study was conducted at Kilimanjaro Christian Medical Center, a referral consultant hospital situated in the North Eastern Zone of Tanzania with a bed capacity of over 600. The center is located in Moshi Municipal in the Kilimanjaro region. It provides medical services to a population of approximately 11 million people. Within the Hospital, the ENT department is the only setting with established ear, nose and throat (ENT) services in northern Tanzania.

### 2.3 Study Population

Included were all patients with Otorhinolaryngology problems who attended the ENT clinic during the study period. Inclusion criteria included all patients aged 2 years and above and consent/assent to participate in the study (for children < 18 years, parents or guardians consent on their behalf) while exclusion criteria included all patients who had been on medications antihistamine 3 days and intranasal steroid 1 week before the enrollment into the study.

### 2.4 Study Sample and Sample Size

The sample size was calculated using the sample size formula developed by the Creative Research system and the sample size was 221 patients. A purposive sampling technique was used, all patients attending the ENT clinic were screened for eligibility and consented to participate in the study and those who met the inclusion criteria and agreed to sign the consent form were included in the study. Dependent variables include allergic rhinitis, sinusitis, otitis media with effusion, hypertrophied inferior turbinate and nasal polyp.

### 2.5 Study Procedure

The study included all patients with a clinical diagnosis of rhinitis presenting with two or more recurrent nasal symptoms of excessive sneezing, watery nasal discharge, nasal congestion and itching of the nose and eyes.

Patients were screened and those who met the inclusion criteria were asked to sign a written informed consent form before being enrolled in the study. For children below the age of 18 years assent was obtained from parents/guardians.

Data administered in the questionnaire include: patient characteristics (e.g. age, sex, occupation, area of residency, education), duration of symptoms, nasal symptoms, eye symptoms, age of onset of symptoms, triggering factors, presence or absence of a family history of allergic rhinitis and asthma, presence of co‐morbidity and the effects on quality of life (defined as interference with daily activities and sleep disturbances).

Appropriate examination was done in all patients, where the candidate with nose complaints was examined by the standard anterior rhinoscopy method, using headlight and/or rigid nasal endoscopy and/or Flexible nasoendoscopy and all patients with boggy inferior turbinate which narrowing or completely obscuring nasal cavities were considered to have inferior turbinate hypertrophy and a polypoid mass seen in the nasal cavity was considered to have nasal polyp. All candidates with ear complaints were inspected using otoscopy/pneumatic otoscopy for evidence of eustachian tube dysfunction and otitis media. Sinusitis in atopic patients was defined by clinical presentation of facial pain, blocked nose, thicker and purulent rhinorrhea and/or anosmia (with two major criteria or one major and two minor criteria qualifying for diagnosis of sinusitis) and CT‐Scan of the paranasal sinuses was taken in all suspected respondents (all were adults) for persistent nasal discharge, septal deviation and thickening mucosa and nasal polyps was interpreted by an experienced radiologist.

The diagnosis of patients with allergic rhinitis and associated co‐morbidities was made clinically based on a validated questionnaire derived from international studies of allergic rhinitis i.e. Score For Allergic Rhinitis (SFAR) and International Study of Asthma and Allergies in Childhood (ISAAC) questionnaire where they had satisfactory sensitivity and specificity, in the absence of any allergic test in developing countries, and Score above or equal to 7 was considered positive for allergic rhinitis [12].

### 2.6 Data Analysis Plan

After completion of data collection, data were coded, cleaned and categorized where necessary by SPSS version 22. Descriptive statistics were performed; categorical variables were summarized using frequency and proportion. Numerical variables were summarized using the measure of central tendency with the respective measures of dispersion. Logistic regression was performed to obtain the Odds Ratio (OR) between Allergic rhinitis and a set of explanatory variables. Results were considered statistically significant at a probability value of less than 0.05.

### 2.7 Ethical Consideration

Approval: Ethical clearance to carry out the study was obtained from the Kilimanjaro Christian Medical University College ethical review committee (CREC) with certificate number 2335.

Consenting: Eligible patients were informed about the purpose of the study and were free to choose to participate or not.

All patients whether consented for the study or not, received equal rights to treatment; medical or surgical intervention based on clinical grounds.

## 3 Results

A total of 232 patients with a clinical diagnosis of rhinitis were approached in 6‐month study period. Out of these 221 patients were included in the study, giving a response rate of 96%. The other remaining 11 patients were excluded because they were on antihistamine 3 days and intranasal steroid 1 week prior to enrollment into the study.

### 3.1 Characteristics of the Study Participants

The study included a total of 221 study participants. The median (range) age of the study participants was 18 (2–78) years. A large proportion of the study participants 72 (32.6%) were aged between 6 and 15 years, 111 (50.2%) were females, 140 (63.4%) were residing in urban areas, 75 (33.9%) had secondary education, 113 (51.1%) were students, 161 (72.9%). This is shown in Table 1.

**Table 1:** Socio‐demographic characteristics of the study participants (N = 221).

Characteristics	n (%)
Age (years) (median(range)	18 (2–78)
**Age (years)**	
≤ 5	22 (9.9)
6–15	72 (32.6)
16–25	57 (25.8)
26–35	35 (15.8)
≥ 36	35 (15.8)
**Sex**	
Male	110 (49.8)
Female	111 (50.2)
**Residence**	
Urban	140 (63.4)
Rural	81 (36.6)
**Education**	
None	22 (9.9)
Primary	71 (32.1)
Secondary	75 (33.9)
Tertiary	53 (23.9)
**Occupation**	
Employed	42 (19.0)
Self‐employed	31 (14.0)
Students	113 (51.1)
Not employed	35 (15.9)

### 3.2 Prevalence of Allergic Rhinitis Among Patients Attending at Ent Department at KCMC

The prevalence of allergic rhinitis among patients with a clinical diagnosis of rhinitis was 53 (23.9%), whereby 32 (60.4%) were male and 21 (39.6%) were female. The median (range) age of the study participants was 18 (2–78) years.

The majority of participants 29 (54.7%) were aged between 6 and 15 years, 32(60.4%) were males, 36 (67.9%) were living in urban areas, 32 (60.4%) had primary education, 38 (71.7%) were students and 39(73.6%) had symptoms duration ranging between 2 and 4 years. This is shown in Table 2.

**Table 2:** Socio‐demographic factors associated with allergic rhinitis (N = 221).

	Allergic Rhinitis	
Factors	OR (95% CI)	*P*‐value
**Age (years)**		
≤ 5	1	
6 to 15	0.81 (0.31; 0.99)	0.046
16 to 25	0.12 (0.03; 0.40)	0.001
26 to 35	0.12 (0.03; 0.48)	0.003
≥ 36	0.25 (0.07; 0.84)	0.025
**Sex**		
Male	1	
Female	0.56 (0.30; 1.07)	0.078
**Residence**		
Urban	1	
Rural	0.77 (0.39; 1.48)	0.428
**Education**		
None	1	
Primary	1.44 (0.54; 3.85)	0.472
Secondary	0.13 (0.04; 0.44)	0.001
Tertiary	0.31 (0.09; 0.98)	0.046
**Occupation**		
Employed	1	
Self‐employed	0.79 (0.17; 3.60)	0.764
Students	3.75 (1.36; 10.32)	0.01
Not employed	1.85 (0.53; 6.45)	0.334
**Symptoms duration (yrs)**		
≤ 1	1	
2 to 4	0.96 (0.38; 2.43)	0.931
> 4	0.84 (0.25; 2.78)	0.775
**Season**		
January to June	1	
July to December	0.42 (0.04; 4.33)	0.464
Not specific	1.25 (0.39; 3.95)	0.704

Factors such as age OR 0.12, 95% CI (0.03; 0.40), education OR 0.13, 95% CI (0.04; 0.44) and occupation OR 3.75, 95% CI (1.36; 10.32), adenotonsillar hypertrophy OR 4.66, 95% CI (2.21; 9.80) and OME OR 4.11, 95% CI (1.32; 12.83) (*p* = 0.009) were found to be significantly associated with allergic rhinitis as shown in Table 2.

### 3.3 The Associated Co‐Morbidities of Allergic Rhinitis Among Patients Attending the ENT Department at KCMC

**Figure 1:**
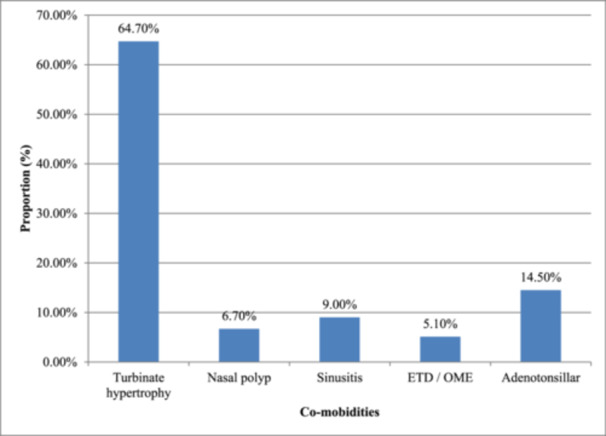
The associated co‐morbidities of allergic rhinitis (*n* = 255).

Turbinate hypertrophy 165 (64.7%) was the leading co‐morbidity of allergic rhinitis among study participants, followed by 37 (14.5%) Adenotonsillar hypertrophy, Sinusitis accounted for 23 (9.0%), Nasal polyp 17 (6.7%) and lastly 13 (5.1%) OME, this is shown in Figure 1.

### 3.4 Correlation Between Adenotonsillar Hypertrophy, Allergic Rhinitis and OME

Adenotonsillar hypertrophy has 2.3 times higher odds of developing OME, while allergic rhinitis has 3.2 times higher odds of developing OME. This is shown in Table 3.

**Table 3:** The association between adenotonsillar hypertrophy, allergic rhinitis and OME.

	OME	
Factors	OR (95% CI)	*p*‐value
Adenotonsillar hypertrophy		
No		
**Yes**	2.31 (0.66; 8.14)	0.193
Allergic rhinitis		
No		
**Yes**	3.25 (0.97; 10.86)	0.055

## 4 Discussion

### 4.1 Prevalence of Allergic Rhinitis

Globally, the prevalence of allergic rhinitis has been increasing, and some environmental factors like pollution have been associated with this [13].

In this study, the prevalence of allergic rhinitis among patients attending the Kilimanjaro Christian Medical Center was 23.9%. This prevalence is analogous to the study done in South Africa and Kinshasa by [14, 15] which was 20% and 23.3% respectively. A slightly lower prevalence was reported to be 14.7% at Bugando Tanzania [16], this could be due to the small sample size and the case definition of the study which was looking mainly at those with co‐morbidities. A higher prevalence of allergic rhinitis has been reported to be 56.7% in Nigeria [17], this was a community‐based study which might have accounted for the higher prevalence. This variation in prevalence from different studies may reflect study selection criteria, differences in measurement techniques and/or survey methods, or diverse geographical sites including differences within regions of the same country [18].

In this study, the majority were children which are comparable with the study done in Nigeria and Bugando Tanzania [16, 19] The reason for the increased number of children with allergic rhinitis to attend medical services may be due to the fact that allergic rhinitis is associated with troublesome symptoms which draw attention of parents to seek immediate medical treatment while a majority of symptoms of allergic rhinitis are being ignored by adult patients.

### 4.2 Co‐Morbidities of Allergic Rhinitis Among Patients Attended During the Study Period

Despite its direct effect on the quality of life, allergic rhinitis has significantly associated co‐morbidities such as sinusitis, otitis media, inferior turbinate hypertrophy, adenoid hypertrophy, middle ear effusion and sinonasal polyps [20]. In this study, inferior turbinate hypertrophy was found in 67.9% of patients, adenotonsillar hypertrophy was present in 35.9%, 13.2% had middle ear effusion, sinusitis and sinonasal polyps were the least complications, which was found in 3.8% and 5.7% of the patients respectively. This study is similar to the study that was done in Muhimbili, Tanzania by [21] in which inferior turbinate hypertrophy was found in 69.4%, adenoid hypertrophy was present in about (30%), 14% had a sinonasal polyp, sinusitis and middle ear effusion had least in co‐morbidities. Also, a study done in the USA [3] found different co‐morbidities that are associated with allergic rhinitis such as depression, anxiety, sleep disturbance and poor school performance. This might be due to the case definition of the study which was mainly looking for social and emotional impact among patients with allergic rhinitis. A study done in South Africa by [11] reported different co‐morbidities in comparison to our study where 58.3% had asthma, 46.0% had eczema and 25.9% had sinusitis. This may be due to the study's case definition, which was mainly looking for an association with other atopic diseases.

Studies have reported that rates of comorbid disorders were lower in children with nonallergic rhinitis than in those with allergic rhinitis, different comorbid were studied where Asthma was reported to be 12.7% for NAR compared to 28% for AR, Eczema 29% for NAR compared to 50% for AR, Conjunctivitis 47.8% for NAR compared to 85.9% for AR, Otitis media 5.8% for NAR compared to 10.3% for AR, Sinusitis 38.2% for NAR compared to 42.8% for AR, Adenoid hypertrophy 25% for NAR compared to 18.2% for AR, Nasal polyps 19.6% for NAR compared to 38.4% for AR [21].

A relation between allergic rhinitis and OME was found but adenotonsillar hypertrophy as a confounder was taken into consideration hence from this study it has been found that adenotonsillar hypertrophy accounts for 2.3 times higher odds of developing OME while allergic rhinitis has 3.2 times higher odds of developing OME and when both conditions exist together may account for higher risk of developing OME.

Generally, in comparison with other studies, we found to have relatively similar prevalence and associated co‐morbidities, taking into account different diagnostic modalities used in different studies, hence this type of diagnostic tool can be implemented and can be used in non‐specialized channels (schools, dispensaries, occupational medicine, etc.) to screen for allergic rhinitis due to its low cost and easy to use.

## 5 Limitations

Further studies on the prevalence and co‐morbidities of allergic rhinitis using different diagnostic modalities in patients attending KCMC are highly recommended. since there might be an overestimation of Allergic Rhinitis using questionnaires only.

## 6 Conclusion

Allergic rhinitis is among the most common health problems affecting Tanzanians with a prevalence of 23.9%. It is a commonly seen disorder in younger age ( < 15 years) which is in correlation with other studies done in Africa and worldwide. Many patients had their first‐degree relatives suffering from the disease, and inferior nasal turbinate hypertrophy was the most common co‐morbidities of Allergic rhinitis.

## Author Contributions

**Kenneth Mlay:** conceptualization, investigation, methodology, visualization, writing–original draft. **Gasper Temba:** investigation. **Adrian Matasha:** investigation. **Pendael Mzonge:** investigation. **Denis Katundu:** data curation, investigation. **Desderius Chussi:** supervision, writing–review and editing.

## Ethics Statement

Ethical approval was obtained from the Kilimanjaro Christian Medical University College Research Ethical Committee with Certificate no. 2335.

## Conflicts of Interest

The authors declare no conflicts of interest.

## Supporting information

Supporting information.

## Data Availability

The authors have nothing to report.
