# Supplementary material for: Allergic Rhinitis and Its Associated Co‐Morbidities Among Patients Attending the ENT Department at Kilimanjaro Christian Medical Center in Northern Tanzania: Cross‐Sectional Study
Source: Immun Inflamm Dis. 2025 Jan 24;13(1):e70130. doi: 10.1002/iid3.70130 (PMC11760977; doi:10.1002/iid3.70130)
Supplement: Supplementary file 1 — Supporting information. [file IID3-13-e70130-s001.docx]

APPENDICES

APPENDIX I: QUESTIONNAIRE ON ALLERGIC RHINITIS QUESTIONNAIRE

Interviewee code:____

**I. SOCIAL DEMOGRAPHIC INFORMATION**

Instructions: Please fill or tick your responses to the right of the following questions.

1. Date of interview:______/______/__________

2. What is your name:______________________________________

3. What is your age:___________ 4. Date of birth: ______/_______/______

(Tick all your answers for the rest of the questionnaire)

5. Sex: ( ) Male ( ) Female

6.Area of residence

a.Urban ( )

b.Rural ( )

7. Educational status

a. No formal education ( )

b. Primary education ( )

c. Secondary education ( )

d. college/university education ( )

8. Occupation

a. Business ( )

b. Teaching ( )

c. Industrial worker ( )

d. Health worker ( )

e. Peasant ( )

f. Student ( )

g. Others ______________________

**II. SPECIFIC QUESTIONS TO ALLERGIC RHINITIS (SCFAR &ISAAC-QUESTIONNAIRE)**

1. When was your first time to experience this illness?________

2. In the past 12 months, have you had any of these problems apart from cold or flu?

a. Sneezing Yes ( ) No ( )

b. Runny nose Yes ( ) No ( )

c. Blocked nose Yes ( ) No ( )

If yes (at least one nose problem) in the past 12 months has this nose problem be accompanied by itchy watery eyes? Yes ( ) No ( )

**In question no 3, refer to the following guidance**

Not at all- You notice the symptoms. A little - They’re easily tolerable.

A moderate amount- Symptoms are hard to tolerate. A lot- sym so bad it’s hard to function.

3. In the past 12 months, how much did this nose problem interfere with your daily activities?

: Not at all ( )

A little ( )

A moderate amount ( )

A lot ( )

4. In which of the past 12 months (or in which season) did this nose problem occur?

Jan ( ) Apr ( ) July ( ) Oct ( )

Feb ( ) May ( ) Aug ( ) Nov ( )

Mar ( ) June ( ) Sept ( ) Dec ( )

No specific period ( ) Others__________

5. What are the triggers factors provoke or increase your nose problem?

a. Dusts ( )

b. Pollens ( )

c. Animals (cat, dogs) ( )

d. Smoke ( )

e. Cold weather ( )

f. Perfume ( )

d. Others (please specify)________________________

6. Do you think to be allergic? Yes ( ) No ( )

7. Have you already been tested for allergy (skin-prick tests to allergens, Ig E)? Yes ( ) No ( )

If Yes: Were they positive? Yes ( ) No ( )

8. Has a doctor already diagnosed that you suffer/suffered from an allergy (asthma, eczema, allergic rhinitis)?

Yes ( ) No ( )

9. Is there anyone in your family (Father Mother Siblings) who suffers from:

a. Asthma Yes ( ) No ( )

b. Eczema Yes ( ) No ( )

c. Allergic rhinitis Yes ( ) No ( )

10. Is there any associated comorbidities

a. turbinate hypertrophy Yes ( ) No ( )

b. Nasal polyp Yes ( ) No ( )

b. sinusitis Yes ( ) No ( )

c. ETD/ OME Yes ( ) No ( )

11. Treatment received

a. Medical Therapy________

b. Partial Resection Inferior Turbinate_____

c. FESS and Nasal Polypectomy______

d. Myringotomy____

APPENDIX: II, LIST OF ABBREVIATIONS

| AR Allergic Rhinitis |
| --- |
| ENT Ear, Nose and Throat |
| FESS Functional Endoscopic Sinus Surgery |
| IL Interleukin |
| IgE Immunoglobin E |
| INS Intranasal Steroid |
| ISAAC International Study of Asthma and Allergies in Childhood |
| KCMC Kilimanjaro Christian Medical Centre. |
| KCMUCo Kilimanjaro Christian Medical University College |
| OME Otitis Media with Effusion |
| ORL Otorhinolaryngology |
| SFAR Score for Allergic Rhinitis |
| Th T helper cell |
| UK United Kingdom |
| SPSS Statistical Package for Social Sciences |
